# Antifungal Activity of Ageritin, a Ribotoxin-like Protein from *Cyclocybe aegerita* Edible Mushroom, against Phytopathogenic Fungi

**DOI:** 10.3390/toxins15090578

**Published:** 2023-09-18

**Authors:** Sara Ragucci, Stefany Castaldi, Nicola Landi, Rachele Isticato, Antimo Di Maro

**Affiliations:** 1 Department of Environmental, Biological and Pharmaceutical Sciences and Technologies (DiSTABiF), University of Campania ‘Luigi Vanvitelli’, Via Vivaldi 43, 81100 Caserta, Italy; sara.ragucci@unicampania.it (S.R.); nicola.landi@unicampania.it (N.L.); 2 Department of Biology, University of Naples ‘Federico II’, Via Cinthia 26, 80126 Naples, Italy; stefany.castaldi@unina.it; 3 Institute of Crystallography, National Research Council of Italy, Via Vivaldi 43, 81100 Caserta, Italy

**Keywords:** ageritin, antifungal activity, phytopathogenic fungi, quinoin, ribosome inactivating proteins, ribotoxin-like proteins

## Abstract

Ageritin from poplar mushrooms is a specific endonuclease that hydrolyzes a single phosphodiester bond located in the sarcin-ricin loop (SRL) of the large rRNA, thereby blocking protein synthesis. Considering the possible biotechnological use of this enzyme, here we report its antifungal activity against virulent fungi affecting crops of economic interest. Our results show that ageritin (200 µg/plug; ~13.5 nmole) inhibits the growth of *Botrytis cinerea* (57%), *Colletotrichum truncatum* (42%), and *Alternaria alternata* (57%), when tested on potato dextrose agar plates. At the same time, no effect was observed against *Trichoderma harzianum* (a fungus promoting beneficial effects in plants). To verify whether the antifungal action of ageritin against *B. cinerea* and *T. harzianum* was due to ribosome damage, we tested ageritin in vitro on partially isolated *B. cinerea* and *T. harzianum* ribosomes. Interestingly, ageritin was able to release the Endo’s fragment from both tested fungal ribosomes. We therefore decided to test the antifungal effect of ageritin on *B. cinerea* and *T. harzianum* using a different growth condition (liquid medium). Differently from the result in solid medium, ageritin can inhibit both *B. cinerea* and *T. harzianum* fungal growth in liquid medium in a concentration-dependent manner up to 35.7% and 38.7%, respectively, at the highest concentration tested (~200 µg/mL; 12 µM), and the analysis of RNA isolated from ageritin-treated cells revealed the presence of Endo’s fragment, highlighting its ability to cross the fungal cell wall and reach the ribosomes. Overall, these data highlight that the efficacy of antifungal treatment to prevent or treat a potential fungal disease may depend not only on the fungal species but also on the conditions of toxin application.

## 1. Introduction

Ageritin is a fungal vacuolar ribonuclease isolated from the fruiting bodies of the edible mushroom *Cyclocybe aegerita* [1]. This enzyme hydrolyzes a specific phosphodiester bond located inside the alpha-Sarcin Ricin loop (SRL; [2]) of large rRNA. Its enzymatic action affects the ribosome structure, impairing protein synthesis. In light of this, ageritin is a member of ribotoxin-like proteins (RL-Ps), a family of specific ribonucleases found in edible mushrooms (basidiomycetes), able to inhibit protein synthesis [1] as previously reported for the ribotoxins protein family, specific extracellular ribonucleases produced by filamentous fungi (ascomycetes) [3,4].

Ageritin has been studied in detail since its discovery in 2017. It is a monomeric protein (~15 kDa) with a high basic pI, no disulfide bonds, and a single reactive cysteinyl residue at the N-terminal region. Moreover, ageritin shows high thermal (~78 °C) and chemical (5.5 M Cm in the presence of guanidine•Cl) stability [5]. In addition, ageritin exhibits a Mg^2+^/Zn^2+^-dependent mechanism [6] and the ability to interact differently with various liposomes, notably with anionic lipids [1].

On the other hand, the determination of the amino acid sequence of both ageritin and ostreatin, the latter well-characterized from *Pleurotus ostreatus*, showed that RL-Ps are structurally analogues to ribotoxins, sharing the same enzymatic target (SRL), whereby these toxin families can be considered both ‘ribosome-targeting toxins’. Furthermore, the screening of commonly consumed edible mushrooms, such as *Boletus edulis*, *Pleurotus eryngii*, and *Agaricus bisporus*, highlights that the RL-Ps family is well distributed among basidiomycetes [1].

Although the biological role of RL-Ps is not yet clarified, ageritin exhibits interesting biological activities in vitro. Indeed, several findings showed that it has: (i) cytotoxic effects against various cancer cell lines; (ii) ribonuclease activity on Tobacco Mosaic Virus (TMV) RNA [1]; (iii) antifungal activity against the green mold *Penicillium digitatum* and the filamentous fungus *Trichoderma asperellum* [1]; and (iv) entomotoxic and nematotoxic activity [7]. In this context, we hypothesized the possible involvement of ageritin in self-defense against predators or in colonization mechanisms necessary to improve the fitness of *C. aegerita* and decided to test the antifungal activity of ageritin against a selected panel of fungi. Therefore, we investigated the antifungal activity of ageritin against virulent fungi infecting plants of economic interest. To achieve this aim, the antifungal activity of ageritin was tested against *Botrytis cinerea*, *Colletotrichum truncatum*, *Alternaria alternata*, and *Septoria nodorum* isolated from tomato, soya, pears, and wheat-infected plants, respectively. Moreover, given the beneficial effect of *Trichoderma harzianum* on plants, both as a growth promoter and as a defense against pathogens [8,9], the effect of ageritin on this fungus was also investigated. Furthermore, considering that fungal susceptibility could be affected by growth conditions, we also evaluated the antifungal activity of ageritin in liquid medium.

In addition, given the availability of quinoin (~29 kDa, basic pI), a type 1 ribosome inactivating protein (RIP) isolated by our group from *Chenopodium quinoa* L. seeds [10], we decided to also test the possible antifungal activity of this enzyme compared to ageritin in an equimolar amount. Indeed, a dose-dependent growth inhibitory effect of quinoin on the plant pathogens *Pseudomonas syringae pv. phaseolicola* and *P. syringae pv. actinidiae* has been demonstrated [10].

Although this enzyme has the same stem-loop structure (SRL) as ribotoxins and RL-Ps, it is an rRNA N-glycosylase (E.C.: 3.2.2.22) [11]. RIPs are toxins, mainly found in plants (angiosperms) [12,13], able to depurinate a single adenine (A_4324_ in rats) and irreversibly block protein synthesis, leading to cell death by the apoptotic pathway [14,15,16]. RIPs are likely to be involved in plant self-defense mechanisms [17], and display a variety of in vitro antimicrobial activities [18] such as antifungal, antibacterial [19] and antiviral properties [20].

Overall, our results show that, unlike quinoin, ageritin could be used as a broad-spectrum biopesticide able to inhibit the growth of most fungi tested.

## 2. Results and Discussion

### 2.1. Ageritin and Quinoin Isolation

**Figure 1:**
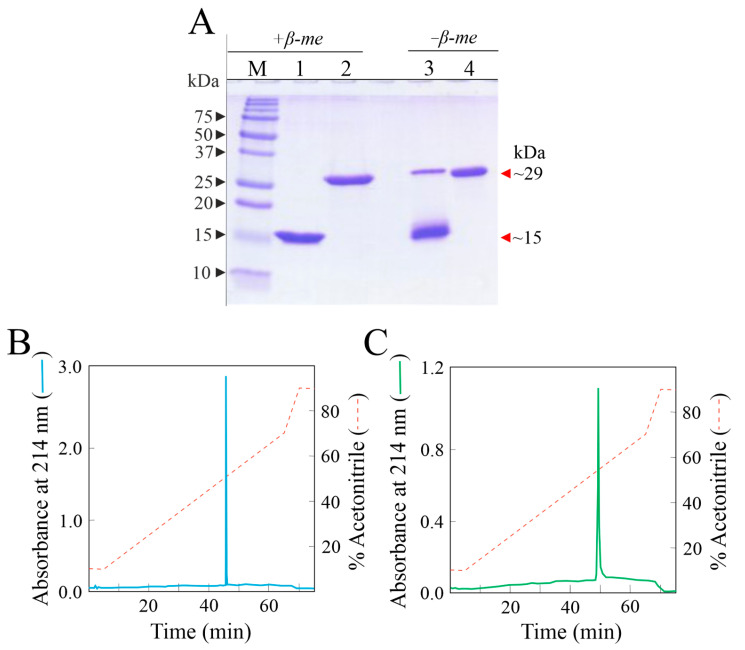
(**A**) SDS-PAGE in a 12% polyacrylamide gel of ageritin and quinoin with (+β-me) and without (−β-me) reducing agents Lane M, molecular markers; lanes 1–3 and 2–4, 3.0 µg of ageritin and quinoin, respectively. (**B**,**C**), RP-HPLC chromatographic profiles of ageritin (50 µg) and quinoin (50 µg), respectively, by using the C-4 analytical column.

Ageritin (ribotoxin-like protein; ~15-kDa) and quinoin (type 1 ribosome inactivating protein; ~29-kDa) were purified from the fruiting bodies and the seeds of *C. aegerita* e *C. quinoa*, respectively, as previously reported [1,10]. The purity of both enzymes was verified by SDS-PAGE and RP-HPLC analyses (Figure 1). In particular, ageritin, in the absence of reducing conditions (lane 3, Figure 1), showed a dimeric form only under SDS-PAGE denaturing conditions due to the single reactive cysteinyl residue [1].

### 2.2. Antifungal Activity of Ageritin and Quinoin in Solid Medium

**Figure 2:**
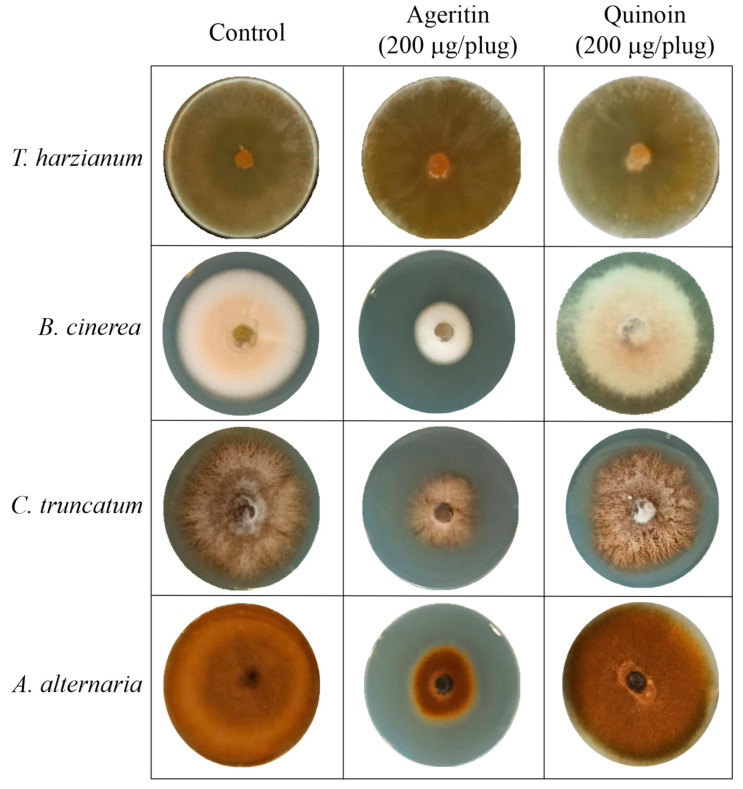
Antifungal assays of ageritin and quinoin against different phytopathogenic fungi in vitro effect of ageritin and quinoin on the mycelial growth of *T. harzianum*, *B. cinerea*, *C. truncatum*, and *A. alternaria*. Representative photographs of mycelia growth inhibition by ageritin and quinoin Only results obtained with 200 µg/plug (~13.5 nmole ageritin or ~7.0 nmole quinoin) are reported. The data are representative of at least three independent experiments, each performed with triplicate samples.

The antifungal activity of ageritin and quinoin was evaluated on potato dextrose agar (PDA) plates as inhibition of mycelial radial growth. To this end, different protein amounts (from 50 to 200 µg/plug; from ~3.0 to 13.3 nmole) were tested against various phytopathogenic model fungi (Table 1). However, an inhibitory effect was observed only using the higher amount of enzyme (Figure 2).

In particular, ageritin was able to inhibit the growth of *B. cinerea*, *C. truncatum*, and *A. alternata* up to about 50% (57%, 42%, and 57%, respectively), while only a slight inhibitory effect against *C. truncatum* was observed in the presence of quinoin (Figure S1). In addition, neither ageritin nor quinoin showed effects against *S. nodorum*. Due to the different molecular weights of ageritin (~15-kDa) and quinoin (~29-kDa), the latter was also tested against the different fungi at 400 µg/plug (13.5 mole), resulting in the latter being inactive. Finally, both enzymes did not inhibit the negative control, *T. harzianum*, under the experimental conditions used.

### 2.3. Antifungal Activity of Ageritin in the Presence of Chitinolytic Enzyme

To investigate ageritin’s antifungal mode of action, a combination of chitinase (a chromatographically purified enzyme from *Streptomyces griseus*) and ageritin was tested against *T. harzianum* and *B. cinerea*, the latter being the most responsive fungus. Chitinases are enzymes capable of hydrolyzing the N-acetylglucosamine polymer chitin, which constitutes the fungal cell wall [25]. These enzymes are expressed in different plant tissues and are implicated in plant resistance against fungal pathogens [26].

**Figure 3:**
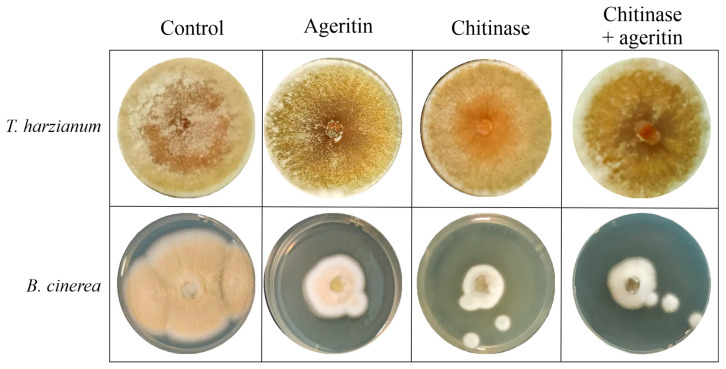
Antifungal activity of ageritin alone and in combination with the chitinase enzyme. Representative photographs of *T. harzianum* and *B. cinerea* growth inhibition by ageritin 200 µg/plug (~13.5 nmole) and chitinase (1-U) alone or in combination The data are representative of at least three independent experiments, each performed with triplicate samples.

To achieve this aim, 1.0 U of chitinase was tested against *T. harzianum* and *B. cinerea* (Figure 3).

As shown in the graphs (Figure S2), the chitinase enzyme is inactive against *T. harzianum*, whereas it inhibits *B. cinerea* growth by approximately 60%. Moreover, a synergistic inhibitory effect was not observed when ageritin (200 µg/plug; ~13.5 nmole) and chitinolytic enzyme were concurrently applied, suggesting that the two molecules have a non-complementary mode of action.

### 2.4. Susceptibility of T. harzianum and B. cinerea Ribosomes to Ageritin and Quinoin

**Figure 4:**
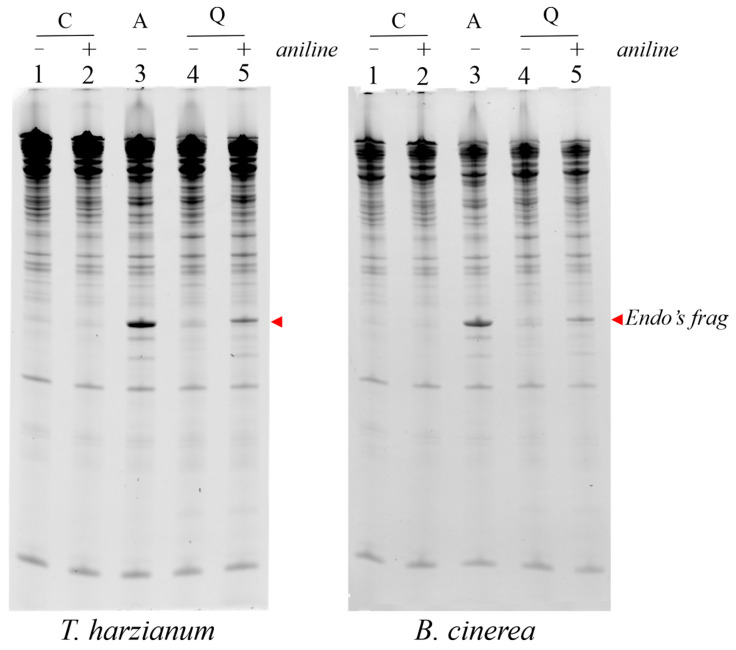
In vitro ribonucleolytic activity of ageritin and quinoin against *T. harzianum* or *B. cinerea* ribosomes. Each lane contained 3.0 µg of RNA isolated from untreated (C), 5.0 μg of ageritin-treated (A), or 5.0 μg of quinoin-treated (Q), *T. harzianum* (**left panel**) or *B. cinerea* (**right panel**) ribosomes. The arrow indicates the fragment (Endo’s fragment) released as a result of ribotoxin action. Samples were treated (+) or not (−) with acid aniline.

In order to ascertain whether the different growth inhibitory effect of both ageritin and quinoin on the tested fungi was a consequence of the different susceptibility of fungal ribosomes to these toxins, we incubated the partially extracted ribosomes (named S30 [27]) obtained from both *T. harzianum* and *B. cinerea* (the latter representative of phytopathogenic fungi) in the presence or absence of the two toxins to detect the release of α-fragment (RNA fragment diagnostic of RL-Ps rRNA endonuclease activity) or β-fragment (RNA fragment diagnostic of RIPs N-glycosylase activity after aniline treatment) in the presence of ageritin or quinoin, respectively [1,10]. Interestingly, as shown in Figure 4, both toxins released the characteristic diagnostic fragment in vitro.

These results highlight that the different toxicity of ageritin and quinoin is likely due to their different abilities to enter the fungal cells and damage ribosomes, leading to the inhibition of protein synthesis and the induction of cell death [5].

### 2.5. Antifungal Activity of Ageritin and Quinoin in Liquid Medium

Several experimental results have shown that fungi undergo morphological and biochemical changes in both the cell wall and membrane depending on growth conditions (i.e., solid or liquid media) [28,29,30]. Indeed, many factors can affect the outcome of in vitro susceptibility tests, including the organism’s inoculum size, incubation time and temperature, growth medium, protein membrane and secreted proteins (e.g., hydrolytic enzymes) and secondary metabolites, as well as diffusion, biochemical stability, or protease susceptibility of biomolecules [30,31,32].

**Figure 5:**
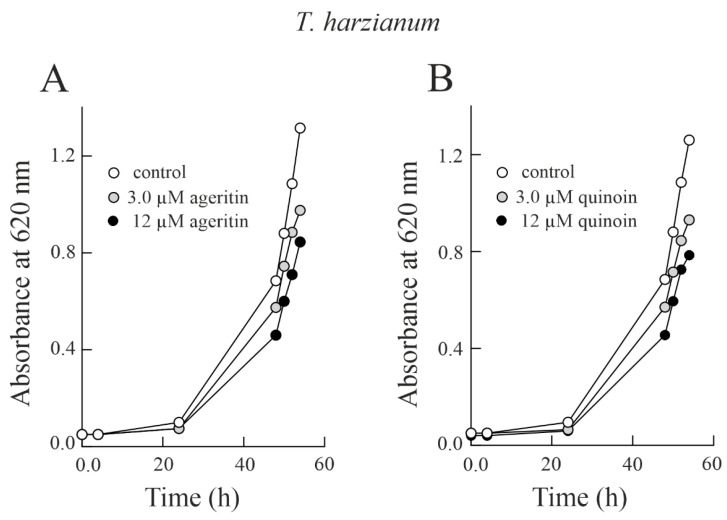
Antifungal activity of ageritin against *T. harzianum* or *B. cinerea* in liquid medium (**A**,**B**), conidia of *T. harzianum* or *B. cinerea*, respectively, were grown at 26 °C in PDB medium in the presence or absence of two different concentrations of ageritin added at 0 h. Fungal growth was measured as an increase in absorbance at 620 nm. The data are representative of at least three independent experiments, each performed with triplicate samples.

In light of this, considering that ageritin and quinoin are able to damage both *T. harzianum* and *B. cinerea* ribosomes (Figure 4), we decided to test the antifungal effect of the two toxins in liquid medium. To this end, we used the minimum and maximum doses previously tested in solid medium in an equimolar amount. Therefore, conidia of *T. harzianum* were grown in PDB medium in the presence of two different concentrations of both ageritin and quinoin for about 54 h. Different from the results obtained by testing the antifungal activity of ageritin and quinoin in solid medium, in this case both toxins are able to reduce the fungal growth in a concentration-dependent manner (Figure 5). However, the higher dose of ageritin against *B. cinerea* showed an efficacy 1.6-fold lower in liquid medium compared to the results obtained in solid medium (Figure 2 and Figure S1). In particular, *T. harzianum* treated with 3.0 and 12 µM ageritin resulted in 25.9% and 35.7% growth inhibition, respectively, after 54 h of growth. Similar results were obtained by exposing the fungus to 3.0 and 12 µM quinoin (corresponding to ageritin equimolar amounts), with 26.2% and 37.7% growth inhibition, respectively.

**Figure 6:**
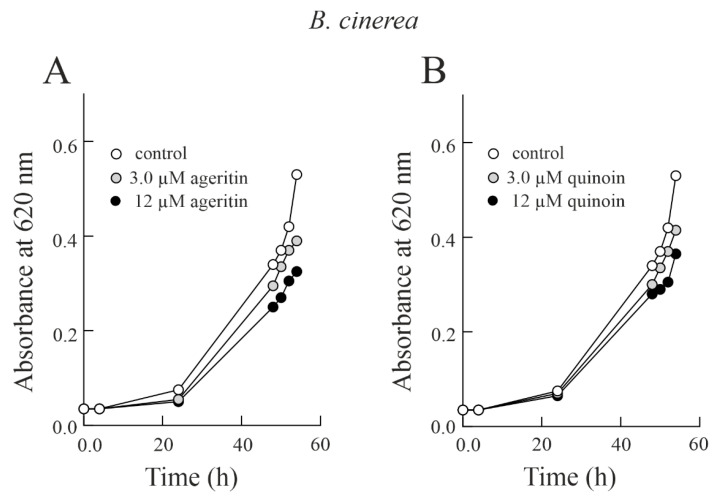
Antifungal activity of quinoin against *T. harzianum* or *B. cinerea* in liquid medium (**A**,**B**), conidia of *T. harzianum* or *B. cinerea*, respectively, were grown at 26 °C in PDB medium in the presence or absence of two different concentrations of quinoin added at 0 h. Fungal growth was measured as an increase in absorbance at 620 nm. The data are representative of at least three independent experiments, each performed with triplicate samples.

The same experiment conducted on the fungus *B. cinerea* (Figure 6) showed similar results. Indeed, *B. cinerea* treated with 3.0 and 12 µM ageritin resulted in 26.4% and 38.7% growth inhibition, respectively, after 54 h of growth, while the same quinoin concentration led to 21.7% and 31.1% growth inhibition, respectively.

**Figure 7:**
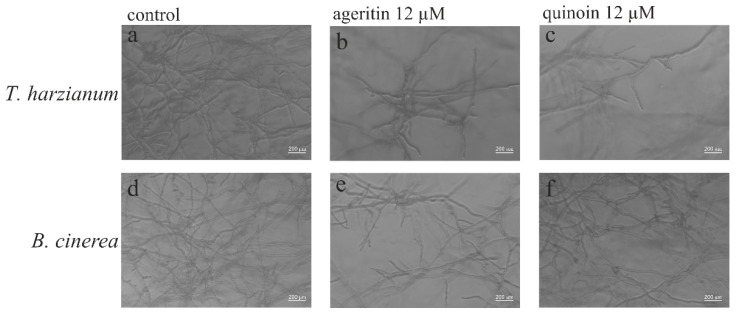
Morphological changes in *T. harzianum* (upper panels) or *B. cinerea* (lower panels) mycelia exposed to ageritin (**b**,**e**) or quinoin (**c**,**f**) The mycelia were grown in the absence (control, (**a**,**d**)) or presence of 12 µM toxins. After incubation for 24 h, samples were visualized using light microscopy at 200× magnification (scale bar: 200 µm). Representative photographs of two wells for each treatment or control are shown.

In addition, the effect of ageritin and quinoin on both *T. harzianum* and *B. cinerea* mycelia growth in liquid medium was also visualized microscopically (Figure 7). The analysis by using light microscopy revealed alterations of hyphal morphology and lower hyphal density after 24 h exposure of both fungi to 12 µM ageritin (Figure 7b,e) or 12 µM quinoin (Figure 7c,f) with respect to the control (Figure 7a,d).

Indeed, while untreated mycelia developed regular and homogeneous hyphae as well as higher hyphal density, mycelia treated with both toxins produced hyper-branching and/or aborted hyphal branches. These data agree with those previously reported for ageritin tested in liquid medium on both *Trichoderma asperellum* and *Penicillium digitatum* [1].

**Figure 8:**
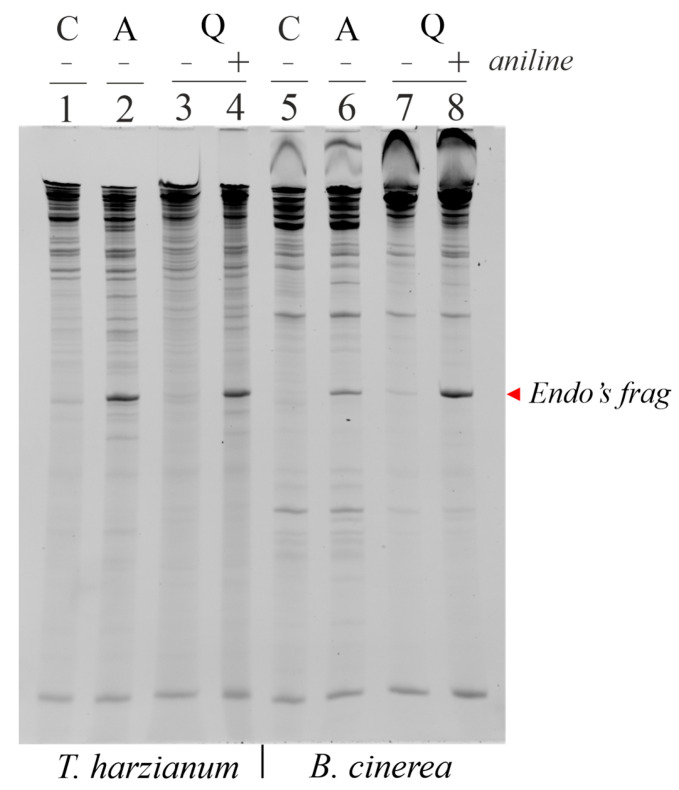
Ribonucleolytic activity of ageritin and quinoin against *T. harzianum* or *B. cinerea* The fungi were grown at 26 °C in PDB in the absence or presence of 12 μM toxins. After incubation for 72 h, the mycelia were extensively washed with sterile water and harvested to extract the RNA. Each lane contained 3 μg RNA isolated from either untreated (C) or 12 μM ageritin-treated (A) or 12 μM quinoin-treated (Q) cultures from *T. harzianum* or *B. cinerea*. The arrow indicates the RNA fragment (Endo’s fragment) released as a consequence of toxin action. Samples were treated (+) or not (−) with acid aniline.

Finally, to determine whether the antifungal effect of RL-P ageritin and type 1 RIP quinoin was a consequence of their ability to inactivate ribosomes, a new set of experiments was carried out in which both fungi were incubated in the presence or absence of 12 µM toxins. To this end, after 3 days of incubation, mycelia were harvested, and total RNA was extracted to visualize the release of the diagnostic Endo’s fragment. Interestingly, as shown in Figure 8, this fragment was absent in both untreated mycelia (Figure 8, lanes 1 and 5) and quinoin-treated mycelia (Figure 8, lanes 3 and 7). On the other hand, both ageritin-treated mycelia (Figure 8, lanes 2 and 6) and quinoin-treated mycelia, the latter after aniline-RNA pretreatment (Figure 8, lanes 4 and 8), are able to release this fragment, confirming that both toxins are able to cross the mycelia cell wall, whereby they reach the fungal ribosomes and exert their specific ribonuclease or N-glycosylase activity.

Overall, these data highlight that the different growth conditions (i.e., solid or liquid medium) can affect the ability of ageritin and quinoin to enter and kill fungal cells, suggesting that not only the fungal species but also the conditions of toxin administration are important for the efficacy of the antifungal treatment.

## 3. Conclusions

Organisms synthesize molecules (secondary metabolites and/or specific proteins or enzymes) necessary to increase their ability to colonize new environments and defend themselves against predators and parasites, thereby increasing their fitness and survival. To achieve this aim, fungi and plants also produce metabolites and proteins as they are unable to escape the predator/parasite (sessile organisms). On the other hand, most of these biomolecules can be considered potential biotechnological tools for biological control, providing a valuable alternative to chemical pesticides.

In this scenario, a potential novel biotechnological tool could be ageritin, a specific ribonuclease isolated from the poplar edible mushroom *C. aegerita*. Indeed, previous studies have already demonstrated the antipathogenic effect of ageritin against different animal and fungal eukaryotic cells.

In this work, we have found that ageritin is able to inhibit the growth of some phytopathogenic fungi affecting crops of economic interest (i.e., *B. cinerea*, *C. truncatum*, and *A. alternata*). Interestingly, the antifungal effect of ageritin depends on the fungal growth conditions (solid or liquid medium). In addition, our results show that ageritin retains its ability to damage fungal ribosomes, highlighting that its efficacy in inhibiting fungal growth is likely due to its ability to penetrate fungal hyphae or act in different growth conditions. Finally, similar experiments were carried out in the presence of quinoin, a type 1 ribosome-inactivating protein isolated from the seeds of *C. quinoa*, which has a similar ability to inhibit fungal growth only in liquid medium, although this enzyme has the same substrate as ageritin, the ribosome.

Further experiments in in vivo plant models and some toxicity tests will be needed to assess the activity of these toxins and their environmental hazards.

## 4. Materials and Methods

### 4.1. Materials

Materials for chromatography have been described elsewhere [10]. All other reagents and chemicals were of analytical grade (Merck Life Science S.r.l., Milan, Italy). Nuclease-treated rabbit reticulocyte lysate system was purchased from Promega (Madison, WI, USA). Chitinase from *Streptomyces griseus* (product number Sae0158) was purchased from Sigma-Aldrich solutions (Merck Life Science S.r.l.).

### 4.2. Ageritin and Quinoin Purification

Ageritin and quinoin were purified according to previously reported procedures [1,9]. Briefly, raw extracts from *C. aegerita* fruiting bodies or *C. quinoa* seeds were acidified with acetic acid and subjected to two consecutive chromatographic steps: Streamline SP (Cytiva, Bucinasco (MI), Italy) step-wise; gel-filtration by Sephadex G-75 Hi-load 26/60 column (Cytiva) on an Akta purification system (Amersham Pharmacia; Milan, Italy). Finally, the last step for ageritin purification is a low-pressure cation exchange chromatography step on a SP-Sepharose column (Cytiva) eluted with a NaCl gradient, while the last step for quinoin purification is a low-pressure cation exchange chromatography step on a CM-Sepharose column (Cytiva) eluted with a NaCl gradient.

Fractions corresponding to main peaks of ageritin or quinoin with inhibitory activity on cell-free protein synthesis were checked by SDS-PAGE analysis, pooled, dialyzed against water, freeze-dried, and stored at −20 °C until use.

### 4.3. Biochemical Analytical Procedures

The general methodology used for analytical biochemical characterization (SDS-PAGE [33] and protein concentration by bicinchoninic acid (BCA) assay protein concentration by bicinchoninic acid (BCA) assay [34] has been described previously [10]. The purity of ageritin or quinoin was checked by RP-HPLC [35] using a BioBasic-4 (150 mm × 4.6 mm, 5-μm particle size; Thermo Fisher Scientific, Waltham, MA, USA) at 25 °C [10]. The following solvents were used: solvent A, Milli-Q water containing 0.1% TFA; solvent B, acetonitrile containing 0.1% TFA. Protein elution was performed using a linear gradient of solvent A and solvent B, from 5% to 65% of solvent B over 60 min at a flow rate of 1.0 mL/min, monitoring the absorbance at 214 nm.

### 4.4. Fungal Cultures

The fungal strains used in this work are listed in Table 1. All the strains were isolated from naturally decayed fruits or plants in Italy and deposited in the fungal culture collection of the Biology Department of the University of Naples, Federico II, Italy [21,23]. Pure cultures were grown for 5 days at 25 ± 1 °C on PDA (potato dextrose agar) medium consisting of 200 g potato, 20 g dextrose, 20 g agar in 1 L water.

### 4.5. Antifungal Activity Detection of Ageritin and Quinoin

The antifungal activity of ageritin or quinoin was evaluated on potato dextrose agar (PDA) (Difco, Fisher Scientific Italia, Segrate (MI), Italy) as inhibition of mycelial radial growth using the method described previously [21] Briefly, 4 mm × 4 mm diameter mycelial plugs were cut from the edge of actively growing 6-day-old colonies, and one plug was placed in the center of a 5 cm diameter Petri dish with the mycelia in contact with the enzymes to be tested. Different amounts of ageritin (50, 100, and 200 μg/plug; 3.0, 6.0, and 12 nmole) or quinoin (200 and 400 μg/plug; 6.0 and 12 nmole) were dissolved in Milli-Q ultrapure water and applied separately to the top of each fungal plug. Plates were incubated at 25 ± 1 °C for 5 days, and the diameter of the fungi was measured at the end. The inhibition percentage of fungal growth was calculated using the following formula:

% = [((Rc − Ri)/Rc) × 100](1)

where Rc is the radial growth of the fungal pathogen in control Petri plates (cm) and Ri is the radial growth of the fungal pathogen in the Petri plates containing ageritin or quinoin (cm).

The beneficial plant fungus *T. harzianum* and Milli-Q ultrapure water were used as controls. Experiments were performed in triplicate and with three independent experiments.

The same method was used when chitinase from *Streptomyces griseus* (Sigma) 1.0 U was tested alone or in combination with ageritin (200 μg/plug; 12 nmole) against *B. cinerea* and *T. harzianum*.

### 4.6. Production of Conidia

The *T. harzianum* and *B. cinerea* fungi were cultured on potato dextrose agar (PDA) slants for 8–10 days in a 25 ± 1 °C shaker-cooling incubator SKI 8 R (Hydrocal B.V., Nijkerk, The Netherlands). Conidial suspensions of *T. harzianum* or *B. cinerea* were prepared by gently scraping the culture surface with a sterile glass rod after the addition of 2–3 mL of sterile milliQ water [36]. The spore suspension obtained was concentrated by centrifugation, dissolved in sterile 20% glycerol, quantified with a hemacytometer using an Eclipse E100 microscope (Nikon Instruments Inc., Melville, NY, USA), and stored at −20 °C.

### 4.7. Preparation of T. harzianum and B. cinerea S30

30,000 g supernatants (S30; [27]) of *T. harzianum* and *B. cinerea* were extracted from *T. harzianum* or *B. cinerea* mycelia obtained after incubation of a conidial suspension (~6000 sp/mL) in 500 mL potato dextrose broth (PDB) medium at 25 ± 1 °C. After 20 days, the mycelium was harvested by filtration through filter paper under vacuum, extensively washed with sterile water, weighed, and stored at −80 °C. Subsequently, an aliquot of the mycelium (~9 g) was ground in a mortar precooled at −20 °C with alumina (1:2, *w*:*w*) at 4 °C for 20 min. Samples were extracted with one volume of 10 mM Tris•Cl buffer (pH 7.6) containing 10 mM Mg(CH_3_COO)_2_, 10 mM KCl, and 6.0 mM β-mercaptoethanol (TMKB buffer 1×) and centrifuged at 30,000× *g* for 20 min at 4 °C. These supernatants, named S30, were stored at −80 °C until use.

### 4.8. rRNA Ribonucleolytic Activity on T. harzianum and B. cinerea Ribosomes

The rRNA ribonucleolytic activities of ageritin and quinoin were assayed in 200 µL samples of S30 supernatants from *T. harzianum* and *B. cinerea*, incubated with either 5.0 µg ageritin or 5.0 µg quinoin for 1h at 30 °C. After treatment, the RNA was extracted with phenol, treated with 1M aniline acetate (pH 4.5) when necessary, and precipitated with ethanol as previously reported [37].

RNA samples were separated on a 5% (*w*/*v*) urea-polyacrylamide gel, stained with ethidium bromide, and visualized under an ultraviolet lamp using a ChemiDoc™ XRS system (Bio-Rad Laboratories Srl, Segrate (MI), Italy).

### 4.9. Antifungal Activity in Liquid Medium

Growth inhibition assays of ageritin and quinoin against *T. harzianum* and *B. cinerea* were performed in 96-well microtiter plates. Conidia of *T. harzianum* and *B. cinerea* (100 spores/well) were incubated at 26 °C in 150 μL PDB medium in the presence of two different equimolar concentrations (3.0 and 12 µM) of ageritin (50 and 200 µg mL^−1^) or quinoin (100 and 400 µg mL^−1^). Fungal growth was monitored spectrophotometrically using a microtiter plate reader [Thermo Scientific™ Multiskan™ FC Microplate Photometer, Segrate (MI), Italy] and microscopically at the incubation times shown in the figures. Images were observed using a Zeiss LSM 700 laser (Carl Zeiss) scanning confocal microscope from Carl Zeiss Microscopy (Jena, Germany). One representative experiment of three independent experiments performed in triplicate is shown.

### 4.10. Ribosome Inactivation Analysis in T. harzianum and B. cinerea Cultures

Mycelia for RNA extraction were prepared from cultures grown in 12 well plates containing 0.75 mL PDB medium inoculated with 500 spores of *T. harzianum* or *B. cinerea* in the absence or presence of 12 µM ageritin or quinoin. The plates were incubated at 26 °C. After 3 days of fungal growth, the mycelium of both *T. harzianum* and *B. cinerea* was harvested by filtration through filter paper under vacuum, extensively washed with sterile water, weighed, and stored at −80 °C. Each sample was prepared with three wells.

Subsequently, 100 µg of mycelium powder were treated with 1 mL Trizol^TM^ reagent (Thermo Fisher Scientific, Rodano (MI), Italy) to extract total RNA and verify the release of Endo’s fragment. Moreover, to visualize the β-fragment released after quinoin treatment, an aliquot of RNA derived from quinoin-treated mycelium was treated with 1.0 M aniline acetate (pH 4.5) and precipitated with ethanol prior to band detection [37]. Finally, RNA samples (3.0 µg) were subjected to electrophoresis at 15 mA in a 7 M urea/5% (*w*/*v*) polyacrylamide gel for 1 h and 30 min and stained with ethidium bromide [36].

### 4.11. Statistical Analysis

The analysis of pathogenic activity was expressed as the mean of three independent experiments. The error bars reported in the figures show the ± standard errors (SE) of the mean from the three experiments.

## Figures and Tables

**Table 1:** Fungal strains, principal infected plants, and their origin country.

Strain	Infected Plant	Country	References
*Botrytis cinerea*	tomato	Italy	[21,22]
*Colletotrichum truncatum*	soya	Italy	[23]
*Alternaria alternata*	pears	Italy	[21]
*Septoria* *nodorum*	wheat	Italy	[24]

## Data Availability

The data presented in this study are available on request from the corresponding author.

## Supplementary Materials

The following supporting information can be downloaded at: https://www.mdpi.com/article/10.3390/toxins15090578/s1, Figure S1: Graphical representation of data reported in Figure 2.; Figure S2: Graphical representation of data reported in Figure 3.

## Abbreviations

RIPs: Ribosome Inactivating Proteins; RL-Ps, Ribotoxin-Like Proteins; SDS-PAGE, Sodium Dodecyl Sulphate-PolyAcrylamide Gel Electrophoresis; SRL, Sarcin Ricin Loop.
