# Supplementary material for: Antifungal Activity of Ageritin, a Ribotoxin-like Protein from Cyclocybe aegerita Edible Mushroom, against Phytopathogenic Fungi
Source: Toxins (Basel). 2023 Sep 18;15(9):578. doi: 10.3390/toxins15090578 (PMC10535218; doi:10.3390/toxins15090578)
Supplement: Supplementary file 1 [file toxins-15-00578-s001.zip › toxins-2601137-supplementary.pdf]

Supplementary Materials: Antifungal activity of ageritin, ribotoxin-like protein from Cyclocybe aegerita edible mushroom, against phytopathogenic fungi

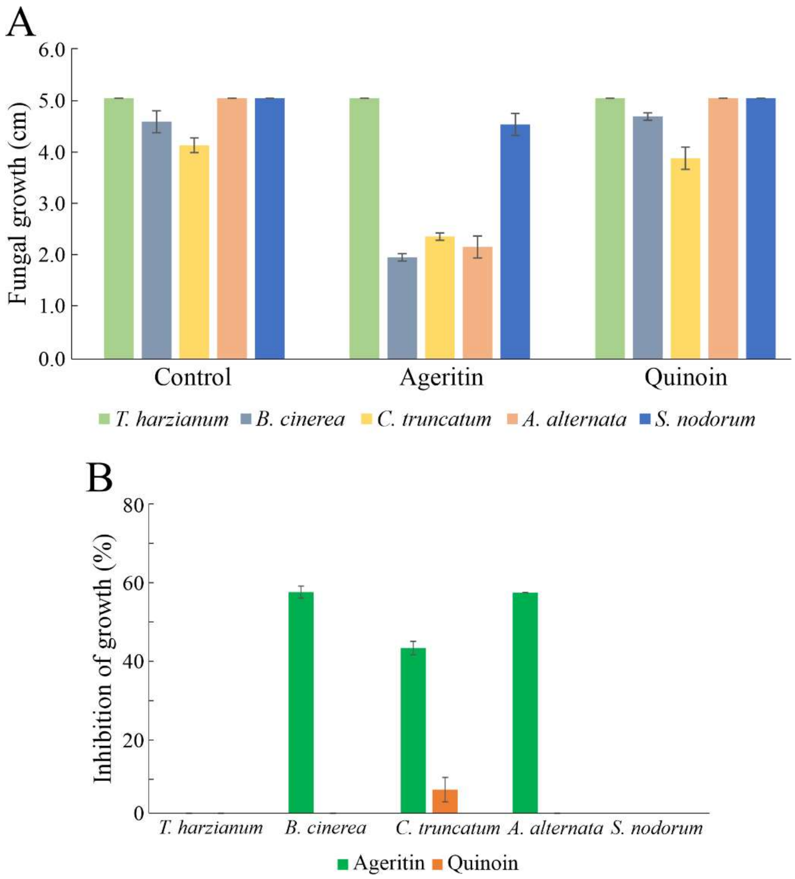

Figure S1. Graphical representation of data reported in Figure 2 (see main text). A) Fungal growth (cm) with and without 200 µg/plug (~13.5 nmole ageritin or ~7.0 nmole quinoin); B) Mycelia growth inhibition (percentage) by ageritin and quinoin tested against different fungi.

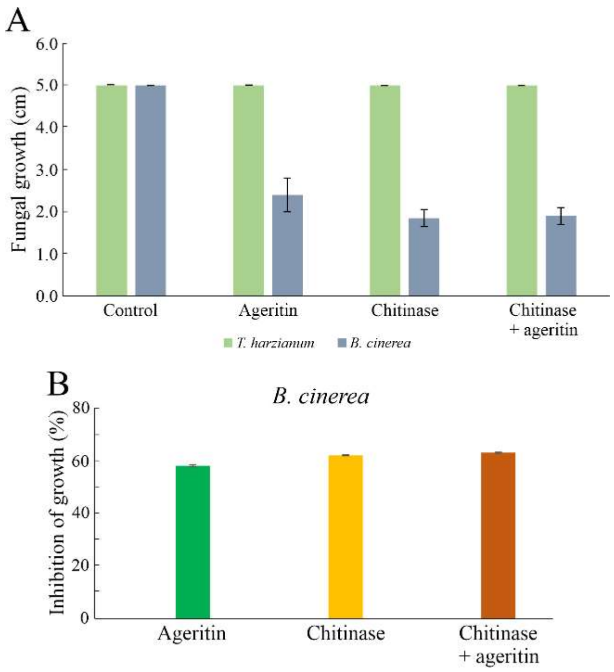

Figure S2. Graphical representation of data reported in Figure 3 (see main text). A) Fungal growth (cm) with and without ageritin and chitinase alone or in combination; B) Grown inhibition (percentage) of B. cinerea.
